# Association of circulating tumor DNA with patient prognosis in surgically resected renal cell carcinoma

**DOI:** 10.1093/oncolo/oyae180

**Published:** 2024-07-16

**Authors:** Andres F Correa, Ekaterina Kalashnikova, Hsin-Ta Wu, Ryan M Winters, Mustafa Balcioglu, Sumedha Sudhaman, Denise C Connolly, Yulan Gong, Robert G Uzzo, Himanshu Sethi, Adam C ElNaggar, Alexey Aleshin, Minetta C Liu, Philip H Abbosh

**Affiliations:** Department of Urology, Fox Chase Cancer Center, Philadelphia, PA, United States; Natera, Inc., Novato, CA, United States; Natera, Inc., Novato, CA, United States; Biosample Repository Facility, Fox Chase Cancer Center, Philadelphia, PA, United States; WuXi Advanced Therapies, Cheltenham, PA, United States; Natera, Inc., Novato, CA, United States; Natera, Inc., Novato, CA, United States; Biosample Repository Facility, Fox Chase Cancer Center, Philadelphia, PA, United States; Nuclear Dynamics and Cancer, Fox Chase Cancer Center, Philadelphia, PA, United States; Department of Pathology, Fox Chase Cancer Center, Philadelphia, PA, United States; Department of Urology, Fox Chase Cancer Center, Philadelphia, PA, United States; Natera, Inc., Novato, CA, United States; Natera, Inc., Novato, CA, United States; Natera, Inc., Novato, CA, United States; Natera, Inc., Novato, CA, United States; Nuclear Dynamics and Cancer, Fox Chase Cancer Center, Philadelphia, PA, United States; Department of Urology, Albert Einstein Medical Center, Philadelphia, PA, United States

**Keywords:** circulating tumor DNA, liquid biopsy, recurrence, renal cell carcinoma

## Abstract

**Background:**

Despite complete resection, 20%-50% of patients with localized renal cell carcinoma (RCC) experience recurrence within 5 years. Accurate assessment of prognosis in high-risk patients would aid in improving outcomes. Here we evaluate the use of circulating tumor DNA (ctDNA) in RCC using banked samples and clinical data from a single institution.

**Methods:**

The cohort consisted of 45 RCC patients (≥pT1b) who underwent complete resection. The presence of ctDNA in plasma was determined using a personalized, tumor-informed ctDNA assay (Signatera RUO, Natera, Inc.). Relationships with outcomes and other relevant clinical variables were assessed. The median follow-up was 62 months.

**Results:**

Plasma ctDNA was detected in 18 out of 36 patients (50%) pre-operatively and was associated with increased tumor size (mean 9.3 cm vs. 7.0 cm, *P* < .05) and high Fuhrman grade (60% grades III-IV vs 27% grade II, *P* = .07). The presence of ctDNA, either pre-operatively or at any time post-operatively, was associated with inferior relapse-free survival (HR = 2.70, *P* = .046; HR = 3.23, *P* = .003, respectively). Among patients who were ctDNA positive at any time point, the sensitivity of relapse prediction was 84% with a PPV of 90%. Of note, ctDNA positivity at a post-surgical time point revealed a PPV of 100% and NPV of 64%. The lack of ctDNA detection at both time points yielded an NPV of 80%.

**Conclusions:**

Detection of plasma ctDNA using a personalized assay is prognostic of recurrence in patients with resected RCC. Herein, we describe a successful approach for its application and identify potential limitations to be addressed in future studies.

## Teaser

Despite complete resection, many patients with localized renal cell carcinoma (RCC) experience recurrence within 5 years. Accurate assessment of prognosis in postoperative patients at high risk for recurrence would aid in improving outcomes. This study evaluated circulating tumor DNA as a biomarker for recurrence using banked samples and clinical data from patients treated with curative intent surgery at a single institution.

**Implications for practice**

Current prediction models of recurrence in surgically resected localized renal cell carcinoma (RCC) are inadequate, limiting their application in clinical decision-making. Herein, we present circulating tumor DNA (ctDNA) as a powerful tool for the prediction of disease recurrence in RCC. The presence of ctDNA before and after surgery was a strong prognostic factor of disease recurrence (HR: 2.7, *P* = .046 and HR: 3.23, *P* = .003). All ctDNA-positive patients post-resection experienced recurrence (12 of 12). This study demonstrates the feasibility of ctDNA to be implemented as a powerful predictor of recurrence in future prediction models and clinical trial eligibility criteria.

## Introduction

Surgery for localized renal cell carcinoma (RCC) is often curative. Clinicopathologic features are used to further define intermediate-high and high-risk disease (30%-50% 5-year recurrence risk), to identify patients who are likely to benefit from adjuvant therapy.^1,2^ A more accurate assessment of prognosis in patients traditionally categorized at a high risk of recurrence is crucial for patient management and clinical decision-making. In RCC, the TNM system remains the standard for risk stratification but lacks accuracy. As a result, several clinicopathological prognostic models have been introduced to enhance risk stratification.^3^ These prognostic nomograms have currently set the standard for clinical guideline development and trial eligibility in RCC.^3^ A recent validation of the aforementioned models demonstrated that the prognostic accuracy of these models is inferior to those originally published, with some models providing a discriminatory capacity no better than a coin flip.^3^ Moreover, the prognostic accuracy of these models degrades over time, limiting their ability to accurately predict late recurrences.^3^

Given the uncertainty provided by current clinicopathological models of recurrence and survival, there is a significant need to develop new prognostic and predictive biomarkers to provide a superior and individualized risk assessment. Circulating tumor DNA (ctDNA) has been evaluated in several solid malignancies and correlates with disease status,^4^ tumor burden,^5^ early identification of recurrence disease,^6-8^ and systemic treatment selection based on the identification of targetable mutations.^9,10^

A robust body of literature supports the use of ctDNA in the early detection of recurrence in asymptomatic patients with various cancer types.^7,11,12^ For instance, in colorectal cancer, ctDNA is an excellent biomarker for recurrence prediction in stages II and III patients after curative-intent surgery, both in the immediate post-operative setting and during follow-up.^7^ Similar findings have been observed in lung and bladder cancer.^6,11^

Reports focusing on detection of ctDNA in RCC are limited. Bettegowda *et al* evaluated 5 patients with RCC in the context of a larger pan-cancer study and showed that ctDNA was detectable in only 2 out of 5 patients.^13^ Other studies have reported ctDNA detection in 50%-91% of metastatic RCC cases and its relationship with disease burden.^14-16^

In this study, we sought to evaluate the use of a personalized and tumor-informed assay in patients with stages I-III, node-negative, RCC or fully resected node-positive and/or metastatic RCC before curative-intent surgery and during surveillance.

## Methods

### Subjects and study design

Whole blood and samples from surgical resection of primary tumor were prospectively collected from 49 stages I-IV renal carcinoma patients through the Fox Chase Biosample Repository (Supplementary Figure S1) under an IRB-approved protocol (IRB#17-9026). According to the protocol, patients were selected to balance recurrent and nonrecurrent cases. The inclusion criteria included patients who had curative intent surgery with ≥pT1b tumor and at least 1 follow-up blood sample. Recurrence was defined clinically based on cross-sectional imaging studies and/or pathological biopsy or excision, when available. Blood was collected pre-operatively and/or at any time post-operatively. The study was performed in accordance with the Declaration of Helsinki. All patients provided written informed consent.

The primary objective of this retrospective analysis was to assess the association of ctDNA detection with clinical recurrence after treatment of localized RCC and assess whether ctDNA was detectable in patients with surgically treated localized RCC prior to recurrence. Samples (*N* = 1) that failed to pass the QC criteria for tumor-tissue whole-exome sequencing (WES), and lack of complete clinical outcome (*N* = 3) were excluded from analysis. A total of 45 patients with at least 18 months of clinical follow-up after partial or radical nephrectomy performed between 2001 and 2017 were included. All patients had primary tumor tissue, genomic DNA (gDNA), and at least one plasma sample available for analysis. Among these patients, 30 out of 45 (67%) had stages I-II tumors, 10 out of 45 (22%) patients had stages III-IV, and 5 out of 45 (11%) patients had unspecified staging.

#### Personalized ctDNA assay using multiplex-PCR (mPCR)-based NGS workflow

Personalized ctDNA assay was designed as previously described.^12^ Briefly, whole-exome sequencing (WES) was performed on formalin-fixed and paraffin-embedded tumor tissue along with matched normal blood samples from each patient. Somatic single-nucleotide variants (SNVs) present in the tumor but absent in the germline were identified from tissue WES for each patient. Multiplex PCR primer pairs targeting up to 16 tumor-specific variants were designed with an established analytical sensitivity to detect tumor DNA if present in plasma. The average depth of mPCR-NGS-based plasma cfDNA sequencing per target was ~100,000×.^12^

### Statistical analysis

Chi-square was used to analyze the association between ctDNA status and clinicopathological variables. Recurrence-free survival (RFS) was determined based on standard radiologic examination, or death due to RCC. Overall survival (OS) was determined based on death due to any cause. To assess the predictive value of ctDNA status on RFS, Kaplan-Meier method using log-rank analysis was performed. Associations were considered statistically significant at *P* ≤ .05 based on a 2-sided distribution. Statistical analysis was performed using Graph Pad Prism 8 and R Statistical software version 3.3.0.

## Results

### Study population

**Figure 1.**
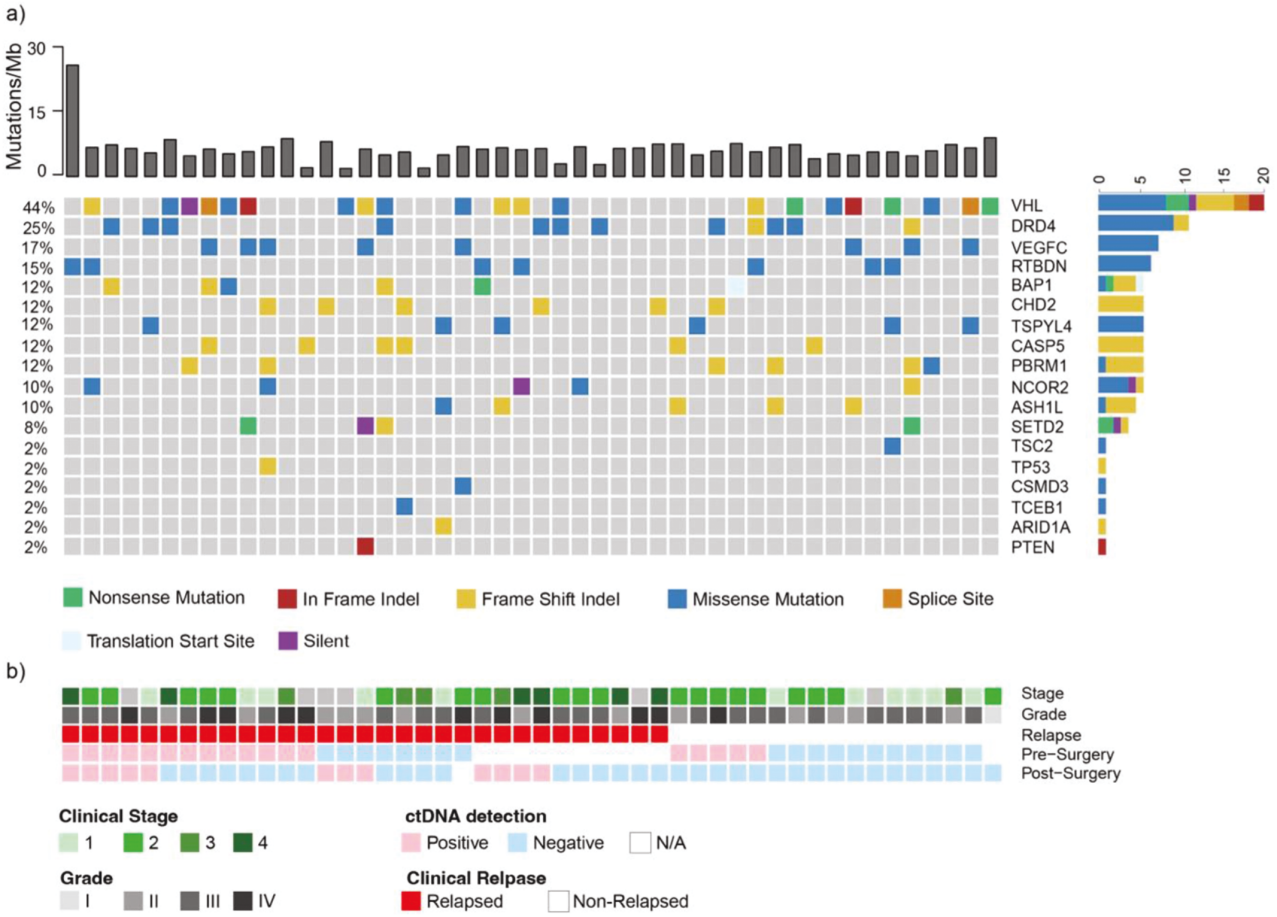
Genetic variants most frequently observed in the patient cohort (*N* = 48) with renal cell carcinoma. (A) Onco‐plot summarizing clinical and genomic features of 48 patients with measurable disease. The top bar graph shows tumor mutation burden (Mutations/Mb) for each patient. The bar graph on the right of the onco-plot represents the mutation frequency of mutation type for each gene in this cohort. Green represents nonsense mutations, red—in frame indels, yellow—frameshift indels, blue—missense mutations, orange—splice site, light blue—translation start site, and purple—silent variants. (B) Clinical characteristics (stage, grade, relapse, and ctDNA detection status pre- and post-surgery).

A total of 45 patients with RCC with clear cell (*N* = 42, 93%), papillary (*N* = 1, 2%), or sarcomatoid variant (*N* = 2, 5%) subtypes with a median follow-up of 62 months (range: 19–219 months) after surgery were included in the study (Table 1). The median age at resection was 61 (range 36-73) years with a median tumor size of 7.5 cm (range 2.9-17 cm; Table 1). Of the 45 patients, 27 (60%) experienced recurrence or death during follow-up. It is notable that the distribution of the most frequent clear cell RCC mutations identified using WES of the studied cohort (Figure 1) was consistent with those reported by The Cancer Genome Atlas (TCGA) consortium,^17-19^ suggesting that this is a representative patient population.

**Table 1.** Characteristics of the patients.

Characteristic	All patients (*N* = 45)
*Sex, n (%)*	
Female	6 (13)
Male	39 (87)
Median age at diagnosis, years (range)	61 (36-73)
Mean tumor size, cm (range)	7.5 (2.9-17)
*Tumor histology, n (%)*	
Clear cell	42 (93)
Papillary	2 (5)
Sarcomatoid	1 (2)
*Clinical stage, n (%)*	
I, II	30 (67)
III, IV	10 (22)
Unspecified	5 (11)
Recurrence, *n* (%)	27 (60)
No recurrence, *n* (%)	18 (40)

### ctDNA detection rates

**Figure 2.**
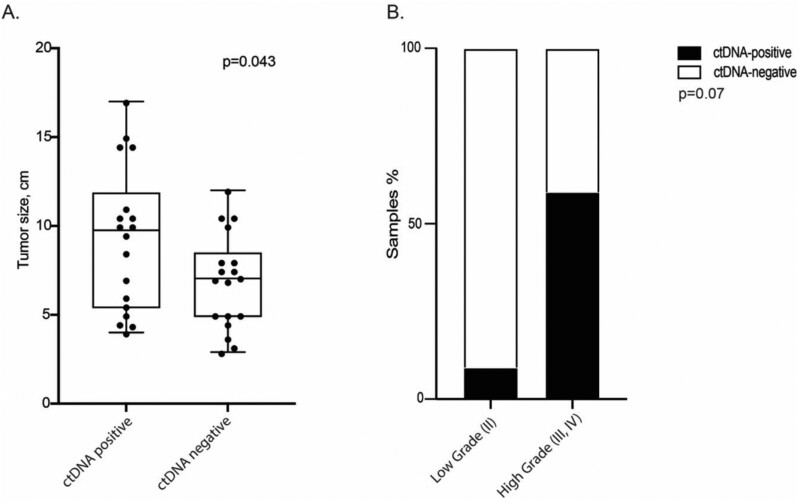
ctDNA status and clinicopathological characteristics. The presence of ctDNA in pre-operative plasma is associated with (A) increased tumor size but not (B) high-grade disease. Chi-square test was used to analyze the association between ctDNA status and clinicopathological variables.

Of 45 patients, 36 had a pre-operative ctDNA time point and 45 had time points during surveillance after surgery. Analysis of plasma samples revealed that 18 out of 36 (50%) patients were ctDNA positive at the pre-operative time point, while 12 out of 45 (27%) had ctDNA detected at any time after surgery (Table 2). The presence of ctDNA in preoperative plasma was not associated with higher grade disease (60% vs. 27%, high [III, IV] vs. low [II], *P* = .07) but was associated with larger size of the primary tumor (9.3 vs 6.9 cm, *P* < .05; Figure 2, Table 2).

**Table 2.** Association of ctDNA status with clinicopathological characteristics.

	Total, *N*	ctDNA-negative, *n* (%)	ctDNA-positive, *n* (%)	Statistics
*Time points*				
Preoperative	36	18 (50)	18 (50)	Not applicable
Postoperative	45	33 (73)	12 (27)	
*Clinical stage*				
Low (I, II)	25	12 (48)	13 (52)	
High (III, IV)	6	3 (50)	3 (50)	*P* = .93
*Grade*				
Low (II)	11	8 (73)	3 (27)	
High (III, IV)	25	10 (40)	15 (60)	*P* = .07
*Average tumor size, cm (range)*				
	8 (2.9-17)	6.9 (2.9-12)	9.3 (4-17)	*P* = .043

### Presence of ctDNA is associated with poor patient prognosis

Preoperatively 50% (18 out of 36) of patients had detectable ctDNA, and among these patients, 72% (13 out of 18) experienced a recurrence within a median of 16.2 (6.1-112.6) months after surgery. ctDNA remained detectable postoperatively in 5 patients, and all of these patients experienced a recurrence within a median of 18.6 (13.5-112.6) months after surgery. Eight relapses were observed in a group of 13 patients with detectable ctDNA at surgery who became ctDNA negative during surveillance. Of 8 patients with recurrence events, only 5 had plasma samples collected within 6 months prior to or at the time of clinical progression (50% event rate).

Among 18 patients with preoperative ctDNA negative plasma samples, 5 relapses were observed (27%). Among those who remained ctDNA negative during surveillance (*n* = 15) a total of 3 recurrence events were observed; however, among patients with events in this group, only one had plasma samples collected within 6 months prior to progression (8% event rate). The remaining 3 patients without ctDNA detection at baseline became ctDNA positive during surveillance, and all of them experienced a recurrence within a median of 31 (17.2-34.1) months after surgery. Lack of blood sampling within clinical proximity to recurrence underscores the difficulty in assessing sensitivity.

**Figure 3.**
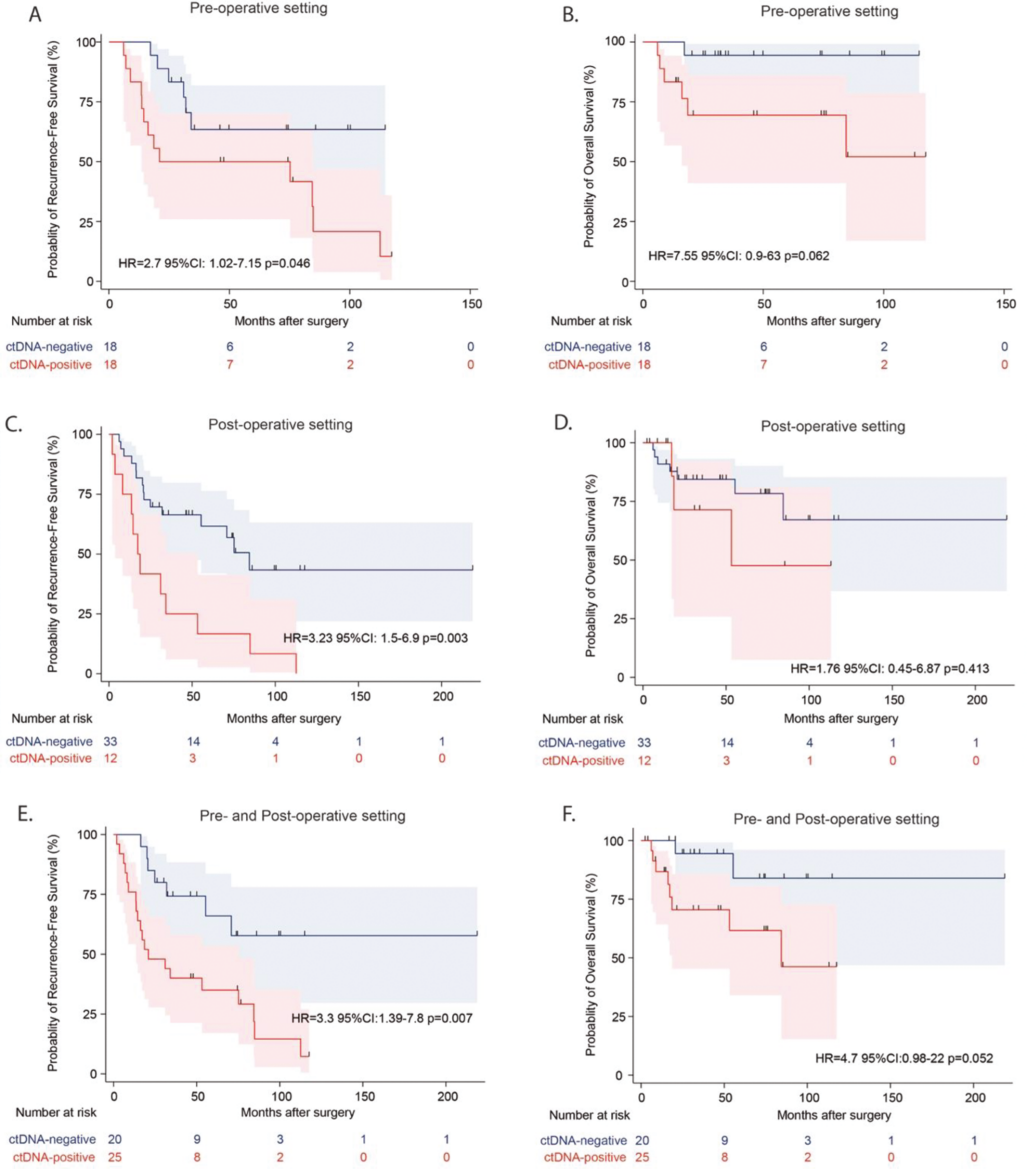
ctDNA status and disease progression. The presence of ctDNA in pre- and post-operative plasma samples is associated with reduced relapse-free survival (A, C) and overall survival (B, D), respectively. (E, F) The presence of ctDNA in plasma samples either prior to primary resection or during surveillance is associated with reduced relapse-free survival and overall survival. Analysis was performed using a log-rank test, and the number of patients at risk at any given time point is indicated.

We explored the prognostic value of ctDNA in this population enriched for recurrence events and found that the presence of ctDNA pre- or any time post-operatively was significantly associated with shorter RFS (HR = 2.7, 95% CI: 1.02-7.15, *P* = .046; HR = 3.23, 95% CI: 1.52-6.98.2, *P* = .00317 respectively; Figure 3A). Additionally in the bivariate analysis, when adjusting for clinical stage, post-surgical ctDNA status remained prognostic (adj. HR: 2.93, 95% CI: 1.25-6.87, *P* = .013), while clinical stage was not (adj. HR: 2.29, 95% CI: 0.96-5.5, *P* = .062). The same trend was observed with ctDNA status before or any time after surgery and OS; however, this was not statistically significant (HR = 7.55, 95% CI: 0.9-63, *P* = .062; HR = 1.62.8, 95% CI: 0.451.7%-6.8711.45%, *P* = .413, respectively). Furthermore, our findings suggest that the presence of ctDNA in plasma samples either prior to primary resection or during surveillance is a strong predictor of reduced RFS (HR = 3.3, 95% CI: 1.39-7.58, *P* = .007) and OS (HR = 4.7, 95% CI: 0.98-22, *P* = .052; Figure 3).

Among patients with 2 time points available, the sensitivity of recurrence prediction based on presurgical samples alone was 68% (13 out of 19) and increased to 84% (19) if ctDNA positivity was observed at either of the time points. Similarly, PPV of a single pre-surgical time point was 72% (13 out of 18). When ctDNA was detected at either of the time points, PPV was 90% (19 out of 21) and was the highest (100%, 12 out of 12) when ctDNA was detected at any time post-surgically. The lack of ctDNA detection before surgery is associated with an NPV of 67% (12 out of 18) and 64% (18 out of 28) after surgery. For those who remain ctDNA negative before and after surgery, NPV rises to 80% (12 out of 15).

## Discussion

ctDNA has the potential to significantly impact cancer management as a minimally invasive, serial testing approach for evaluating molecular residual disease, for monitoring treatment response and disease relapse. Recent studies with Signatera, a clinically validated, tumor-informed 16-plex PCR assay, have shown the capability of utilizing ctDNA as a prognostic marker through the detection of tumor-specific DNA variants in the plasma, signaling persistent disease, well before being detectable by imaging in colorectal, lung, breast, bladder, ovarian, and esophageal cancer.^6,7,11,12^ Here we explore the application of this assay as a novel and powerful prognostic marker of disease recurrence in patients with stage (I-IV) RCC.

Most RCC-focused publications related to ctDNA describe the challenges of detection. Although being highly vascularized, it has been suggested that kidney cancer does not “shed” ctDNA as much as other tumor types.^20^ One hypothesis suggests that the increased infiltration of phagocytes leads to rapid clearance of the cellular debris preventing release into the bloodstream.^21^ Bacon et al showed a 3.9% median fraction of ctDNA in mRCC compared to metastatic prostate (15.3%) or bladder cancer (24.6%).^21^ A more recent study by Peterson et al reported a presurgical detection rate of 81% in mRCC. Despite the fact that our cohort mostly consisted of patients with early-stage, low-risk disease (33 patients ≤ T2, 15 patients with low-grade disease), ctDNA was detectable in 50% of pre-operative samples, compared to previous studies using alternative assays reporting similar detection rates (30%-50%) in mRCC.^13,22-24^ In addition, it is important to mention that the majority of the sample set was limited in sample volume leading to below-expected levels of extracted cfDNA, which could in term have impacted the findings. Despite the limitations of the study, ctDNA was detected in 50% of patients prior to complete surgical resection and was found to be associated with tumor size (*P* = .043); a trending association with grade (*P* = .07) was also observed. Detection of ctDNA at a single time point before surgery was associated with a 72% chance of recurrence. Among patients with 2 time points, detection of ctDNA in at least one of them identified 84% (19) of those with recurrence. ctDNA detection during surveillance served as a dichotomous and unambiguous signal of molecular progression that corresponded to 100% PPV and specificity as all ctDNA-positive patients in the post-surgical setting recurred. Recurrences were seen in 8 patients who were ctDNA negative prior to (<6 months) or at the time of recurrence. Interestingly, 6 patients had presurgical samples available and 5 out of 6 patients had ctDNA detected at surgery. Our results strongly suggest that monitoring ctDNA status serially, including prior to primary resection, allows more accurate identification of patients at high risk of recurrence. This combined analysis of pre- and post-operative timepoints is particularly important for patients with a consistently negative ctDNA result that identifies those with higher likelihood of a favorable outcome, which could be relevant for clinical trial design. Contextualizing this result in other cancer indications, an NPV of 80%-100% is expected,^12,25^ which however is dependent on cancer type and the overall length of follow-up. Although to be best tested in a prospective clinical trial design, this hypothesis may help identify patients who are least likely to have an event/disease progression.

Patients with RCC are in great need of more reliable biomarkers to guide clinical decision-making. Given the absence of any other prognostic biomarkers, ctDNA may be able to guide treatment decision-making. Specifically, decisions regarding active surveillance for primary tumors, adjuvant therapy, and even treatment of metastatic disease.

In summary, we demonstrate that ctDNA may have prognostic value in RCC. Further, ctDNA was detected in half of the patients in our cohort which was comprised of patients with resected disease and was preselected for recurrent and non-recurrent disease. There is therefore a potential role for ctDNA as a prognostic biomarker in the management of RCC.

## Supplementary material

Supplementary material is available at *The Oncologist* online.

oyae180_suppl_Supplementary_Figure_1

## Data Availability

The authors declare that all relevant data used to conduct the analyses are available within the article. Any additional requests pertaining to the study will be reviewed within a time frame of 2 to 3 weeks by corresponding authors to verify whether the request is subject to any intellectual property or confidentiality obligations. All data shared will be de-identified.
