# Supplementary material for: Association of circulating tumor DNA with patient prognosis in surgically resected renal cell carcinoma
Source: Oncologist. 2024 Jul 16;29(10):887–93. doi: 10.1093/oncolo/oyae180 (PMC11449105; doi:10.1093/oncolo/oyae180)
Supplement: oyae180_suppl_Supplementary_Figure_1 [file oyae180_suppl_supplementary_figure_1.docx]

**
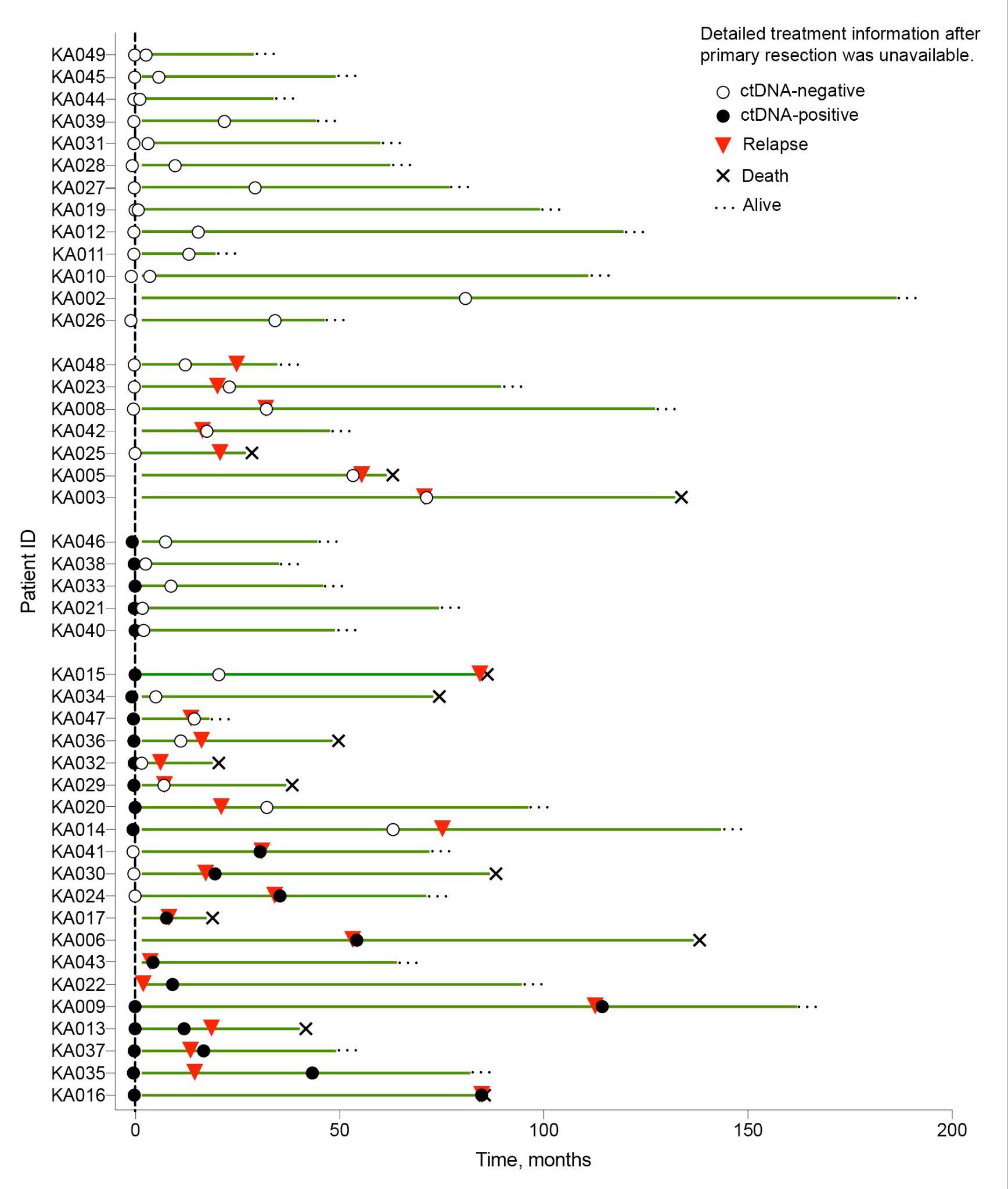
**

***Supplementary Figure 1: Disease recurrence and survival of patients with detected ctDNA.*** *A summary of ctDNA detection in plasma samples (n=81), length of clinical follow-up after surgery (green line), and outcome for each patient (n=45) are shown. ctDNA negative and positive samples are depicted as white and black circles accordingly. Outcomes of patients are shown as red triangles to indicate relapse, an “X” signifies a patient’s death, while three dots represent that the patient was still alive at the last follow-up visit. Due to unavailability of treatment information for all patients, the interpretation of ctDNA results is limited.*
